# First record of the land gastropod genus *Otesiopsis* from South Korea (Helicarionoidea Bourguignat, 1877)

**DOI:** 10.3897/BDJ.7.e46984

**Published:** 2019-11-08

**Authors:** Kazuki Kimura, Satoshi Chiba, Jae-Hong Pak

**Affiliations:** 1 Research Institute for Ulleung-do and Dok-do islands, Department of Biology, Kyungpook National University, Daegu, South Korea Research Institute for Ulleung-do and Dok-do islands, Department of Biology, Kyungpook National University Daegu South Korea; 2 Department of Environmental Life Sciences, Graduate School of Life Sciences, Tohoku University, Sendai, Japan Department of Environmental Life Sciences, Graduate School of Life Sciences, Tohoku University Sendai Japan

**Keywords:** conservation, land snails, insular biodiversity, new records, geographic distribution

## Abstract

*Otesiopsis* Habe, 1946 is a land snail genus, which is known so far from Japan and Taiwan. Individuals of this genus were collected from Gageodo Island, South Korea. This is the first record of the genus *Otesiopsis* from this country. The individuals from Gageodo Island appear to be a new species because of their unique shell morphologies, while the details of their anatomy are still lacking. Further studies are encouraged to investigate the taxonomic and conservation status of the genus *Otesiopsis* in South Korea.

## Introduction

The helicarionid land snail genus *Otesiopsis* Habe, 1946 is known from Japan and Taiwan (Habe 1946). There are five recognised species: *O. japonica* (Möllendorff, 1885), *O. taiwanica* (Kuroda, 1941), *O. kawaguchii* Habe, 1961, *O. kanmuriyamensis* Azuma, 1973 and *O. yamamotoaizoi* Minato, 1974 (Möllendorff 1885, Kuroda 1941, Habe 1961, Azuma 1973, Minato 1974). While *O. japonica* and *O. taiwanica* have relatively wide distribution areas in western Japan and on the entire island of Taiwan, respectively (Otani et al. 2004; Hsieh et al. 2006), the remaining three species seem to inhabit only limited mountain areas or small islets (Habe 1961; Azuma 1973; Minato 1974). The population densities of *Otesiopsis* are considered low (e.g. Takehira et al. 2015). At present, there are only five occurrence records in GBIF (https://www.gbif.org/species/4599174). Due to the low population densities and/or restricted distributions, all species except *O. taiwanica* are listed in the national level red data book (Ministry of Environment 2019).

**Figure 1.**
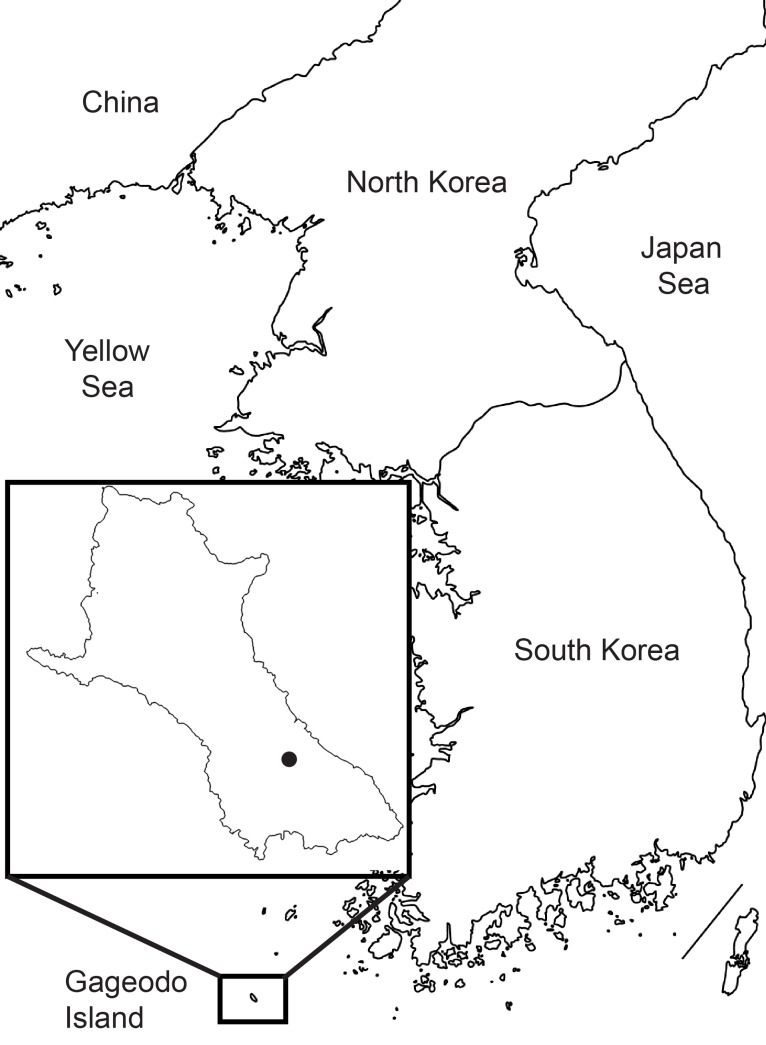
Sample collection locality in Gageodo Island, South Korea (34°03'41.2"N, 125°07'29.5"E).

Gageodo Island is a small island in South Korea, with an area of about 9.1 km^2^ and situated about 120 km from the Korean peninsula (Fig. 1). The highest mountain on the island is Mt. Doksilsan with an elevation of 639 m. The non-marine gastropods have so far not been investigated on Gageodo Island.

## Materials and Methods

On 12-14 June 2019, during a field survey, three specimens of the genus *Otesiopsis* were collected on Gageodo Island, Sinan County, South Jeolla Province (Fig. 1). The shells were examined under a light microscope (Olympus SZ40). The collected specimens were deposited in the Specimen Repository of Kuroha Shell Museum (Voucher No: MNKS3347; 3405; 3476).

## Results and Discussion

Order Stylommatophora Schmidt, 1855 (see Schmidt 1855)

Family Helicarionidae Bourguignat, 1877 (see Bourguignat 1877)

Genus *Otesiopsis* Habe, 1946

*Otesiopsis* sp.

### Description

Shell medium size for this genus, very thin, glossy, dull chestnut brown, with weak growth lines. Whorls 5½ increasing regularly in diameter. Protoconch 1½ whorls with a smooth and glossy surface. Spire moderately low. Suture shallow but distinct. Body whorl with a strong peripheral keel. Base strongly glossy, more convex than the upper part and concave in the umbilical region. Aperture wide, semilunate in outline. Outer lip very thin, sharp, non-reflected, angulated at the periphery, gently curved at the base. Columellar lip short, oblique, slightly thickened. Umbilicus narrowly open.

**Figure 2.**
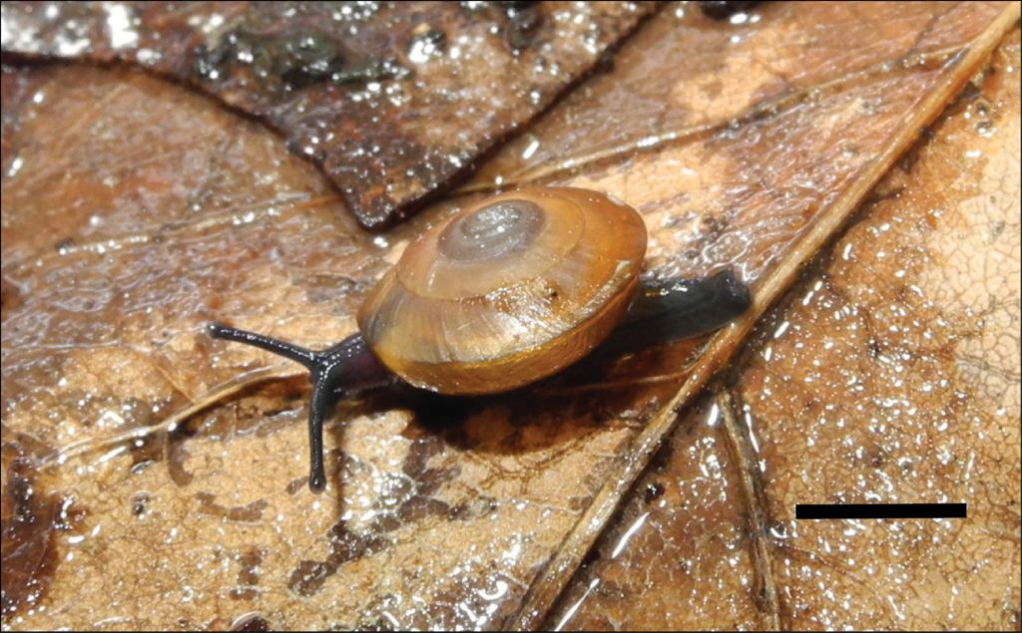
Living individual of *Otesiopsis* collected from Gageodo Island, South Korea. Scale bar = 5.0 mm.

**Figure 3.**
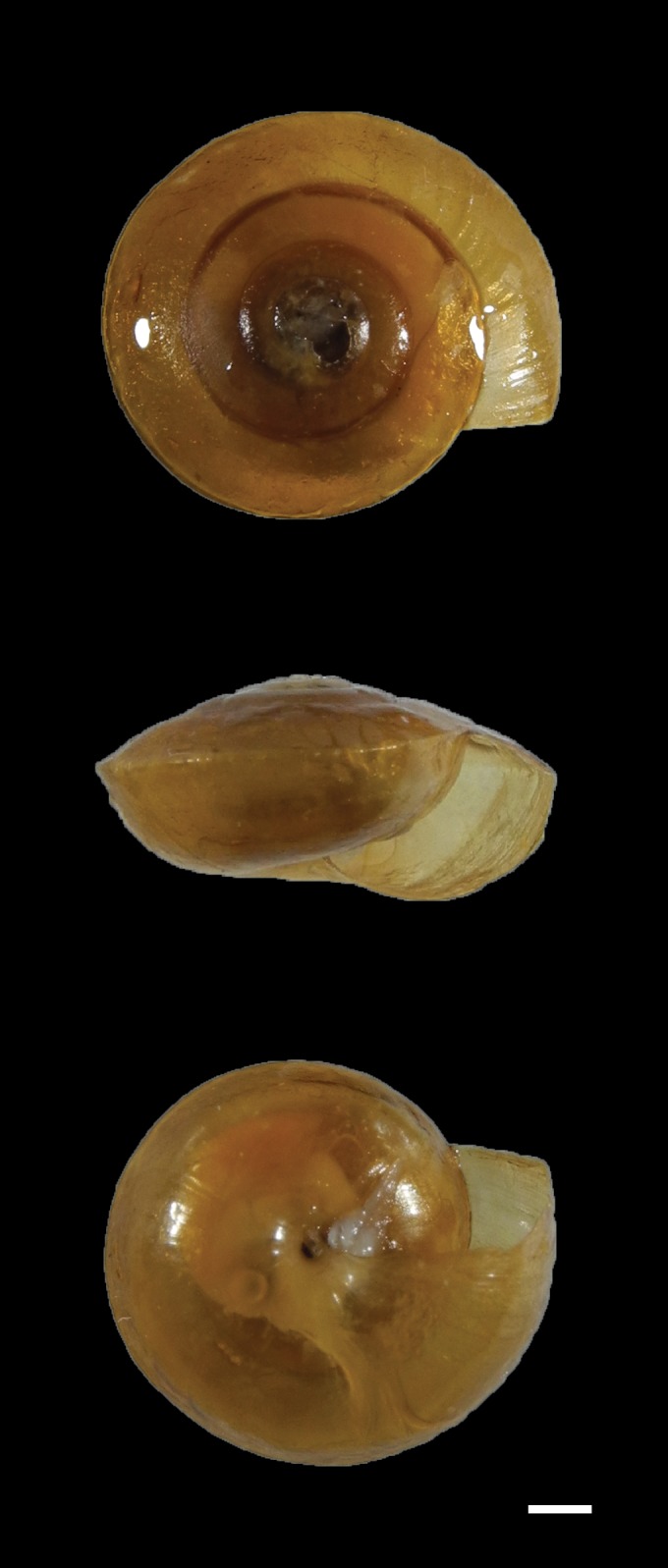
Shell morphology of *Otesiopsis* collected from Gageodo Island, South Korea (Voucher No: MNKS3405). The protoconch was broken. Part of the shell was underexposed. Scale bar = 1.0 mm.

Height 3.64 mm, width 7.24 mm (Figs 2, 3, Voucher No: MNKS3405).

### Remarks

Of the three specimens collected in this study, only one individual was sexually mature, which was confirmed by its egg-laying behaviour. Although intraspecific variation needs to be investigated in the *Otesiopsis* snails on Gageodo Island, the adult specimen examined here exhibited distinct differences from other *Otesiopsis* species. The Gageodo *Otesiopsis* has a smaller body size and thicker shell base than *O. japonica* and *O. taiwanica*. It can be distinguished from *O. kawaguchii* and *O. kanmuriyamensis* by having a perforated umbilicus and from *O. yamamotoaizoi* by a larger body size and carinate periphery. *O. yamamotoaizoi* recorded from the Danjo Islands (Nagasaki prefecture, Japan), situated about 380 km southeast of Gageodo Island, is the geographically closest species of *Otesiopsis*.

The *Otesiopsis* snails occurred from Gageodo Island are likely to be an undescribed species. However, morphologies of its reproductive system and radula, which are important traits for classification of *Otesiopsis* (e.g. Habe 1946; Azuma 1973), were not obtained in this study. Further anatomical studies using additional adult specimens are needed to clarify this newly recorded land snail from South Korea. In addition, since the *Otesiopsis* species are generally rare and vulnerable, further field surveys on Gageodo Island and its adjacent areas would be of importance in order to confirm its conservation status.
